# Lymphatic flow restoration after stripping surgery for varicose veins: A case report

**DOI:** 10.1177/2050313X19849265

**Published:** 2019-05-08

**Authors:** Shuhei Yoshida, Isao Koshima, Hirofumi Imai, Ayano Sasaki, Yumio Fujioka, Shogo Nagamatsu, Kazunori Yokota

**Affiliations:** 1 The International Center for Lymphedema, Hiroshima University Hospital, Hiroshima, Japan; 2 Department of Plastic and Reconstructive Surgery, Hiroshima University, Hiroshima, Japan

**Keywords:** Lymphedema, varicose vein, stripping surgery

## Abstract

It has been suggested that the dynamics of the venous and lymphatic systems interact as a mutually dependent dual outflow system and that derangement of lymph flow could be reversed by surgical treatment of venous incompetence. In this report, we describe a patient in whom lymphatic function was restored after stripping of the great saphenous vein for varicosity. The patient was a 79-year-old woman who had varicose veins along the medial side of an edematous left leg. Lymphatic function was investigated using indocyanine green imaging to evaluate for the presence of lymphedema. Based on the findings, we made a diagnosis of bilateral varicosity of the great saphenous vein with left-sided lymphedema. The great saphenous vein was stripped between the groin and ankle on both sides. At 3 months after the stripping procedure, lymphatic flow was observed immediately after injection of indocyanine green in both legs along the medial side from the foot to the groin. We therefore determined that lymphatic flow had been restored after the stripping surgery. The functions of the venous and lymphatic systems are thought to be closely related, and that, if the function of one declines, the other will also be affected. Treatment of venous system, including stripping, may help to break the vicious cycle of lymphatic stasis and venous insufficiency.

## Introduction

Lymphatic complications, such as lymphocele, lymphatic fistula, and lymphedema, are some of the most common complications after surgery for varicose veins,^1–5^ although their frequency and the risk of long-lasting sequelae are relatively low.^1,6–8^ However, it has been suggested that surgical treatment of varicose veins can normalize lymphatic function.^9^ Furthermore, venous dynamics and lymph dynamics may interact as an inseparable and mutually dependent dual outflow system, and varicose veins could affect lymphatic function and cause slowing of lymphatic flow in the lower limbs. Therefore, surgical treatment of venous incompetence could reverse disturbance of lymph flow.^9^

In this report, we describe a patient in whom return of lymphatic function was confirmed by indocyanine green (ICG) after stripping of the great saphenous vein (GSV) for varicosity.

## Case

**Figure 1.**
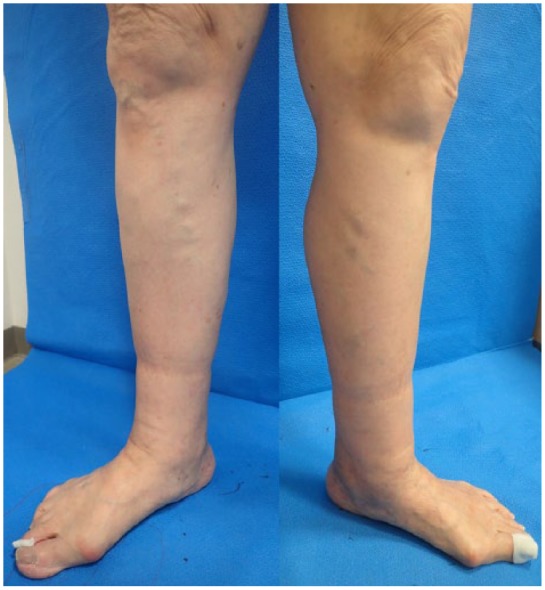
The patient was a 79-year-old woman who had first become aware of edema in her leg several years earlier. She presented to our clinic with a lower leg edema below the knee. Lower extremity lymphedema (LEL) index was 179 on the right and 173 on the left. There were varicose veins along the medial side seen in the edematous leg. Clinical disease severity was graded C3 using the revised CEAP (Clinical, Etiologic, Anatomic, and Pathophysiologic) classification.

The patient was a 79-year-old woman who had first become aware of leg edema several years earlier. She presented to our clinic with edema below the knee (Figure 1). Her lower extremity lymphedema (LEL) index (calculated by summation of the squares of the circumferences for five areas in each lower extremity divided by the body mass index)^10^ was 179 on the right and 173 on the left. There were varicose veins along the medial side in the edematous leg. Her clinical disease severity was graded as C3 using the revised Clinical, Etiologic, Anatomic, and Pathophysiologic (CEAP) classification.^11^

### Color Doppler ultrasound scan

Duplex ultrasound scanning was performed using a 7.5-MHz transducer and the Noblus™ Ultrasound Diagnostic System (Hitachi Aloka, Tokyo, Japan) with the patient in the standing position. Reflux longer than 2.0 s was observed along the entire length of the GSV in both the thigh and lower leg regions after a provocative maneuver using manual compression of the calf. Ultrasonography did not reveal venous thrombosis. The varicose veins were stripped successfully from the ankle to the groin bilaterally (Figure 3).

### ICG images

**Figure 2.**
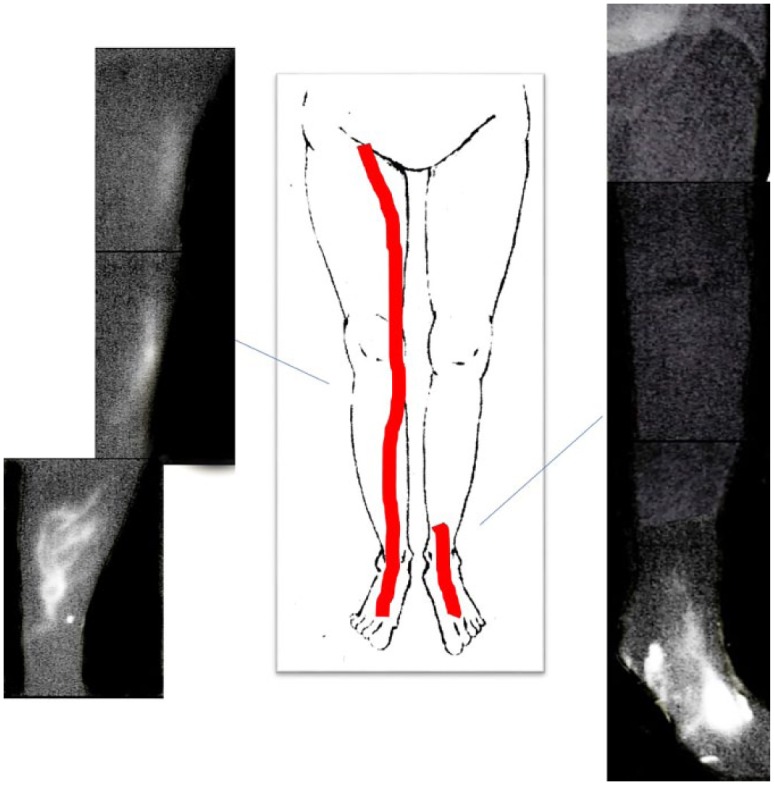
ICG image pre-operation. Lymphatic flow was observed in the right leg along the medial side from foot to groin immediately within 3 min after ICG injection; however, no flow was observed beyond the middle part of the left lower leg even after adding passive movement to the ankle and knee for over 10 min. At 3 h after ICG injection, there were no changes in ICG lymphography. Right leg showed linear pattern, while the left leg showed less enhancement pattern.

Clinical disease severity was graded as stage 2 using the Campisi clinical staging system for lymphedema.^12^ Lymphatic function was investigated to verify lymphedema and to confirm the status of lymphatic flow before and after stripping. The procedure was carried out by injecting 0.1–0.2 mL of ICG dye (Diagnogreen 0.5%; Daiichi Pharmaceuticals, Tokyo, Japan) subcutaneously into the first interdigital space and into the posterior lateral condylar region. ICG images were then acquired using a photodynamic eye system (Hamamatsu Photonics, Hamamatsu, Japan). The patient lay still in the supine position while repeating dorsiflexion and plantar flexion of the toes and ankles at her own pace during measurement of transit time. The purpose of repeating dorsiflexion and plantar flexion of the toes and ankles was to reduce the excessive delay of flow, which often occurs in running flow over the ankle joints. When flow was too slow, passive movement of the ankle or knee was applied to confirm if flow was interrupted or not; this had almost no effect on the results of ICG lymphography. Transit time was defined as the time required for uptake of ICG at the groin.^13^ Lymphatic flow was observed in the right leg along the medial side from the foot to the groin immediately within 3 min of injection of ICG; however, lymphatic flow was blocked and dilatation of the lymphatics was subsequently absent beyond the middle part of the left lower leg even with passive movement at the ankle and knee for over 10 min (Supplement Figure 1). There were no changes in the findings on ICG lymphography in the 3 h after injection of ICG. The right leg showed a linear pattern, but the left leg showed less enhancement pattern (Figure 2).

Based on these findings, we made a diagnosis of bilateral varicosity of the GSV with left-sided lymphedema. Stripping under lumbar anesthesia was planned first with subsequent lymphaticovenular anastomoses.

### Surgical procedure

Ligation and division of the tributaries at the saphenofemoral junction and dissection of the GSV were performed at the level of the ankle. The GSV between the groin and ankle was subsequently stripped on both sides under tumescent local anesthesia to minimize bleeding as much as possible. No perforator veins in the lower leg were divided. Sclerotherapy was not performed perioperatively. Limb compression was performed for one night to prevent postoperative hemorrhage.

## Results

**Figure 3.**
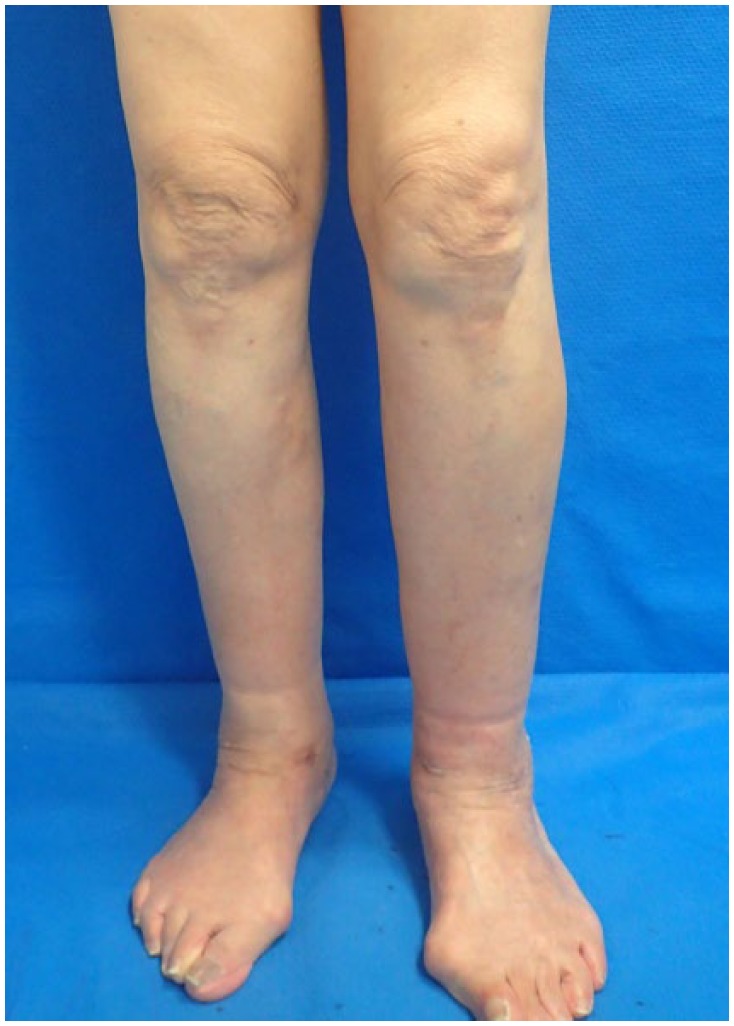
The varicose veins were stripped successfully from ankle to groin bilaterally. LEL index was 163 on the right and 162 on the left.

The varicose veins were stripped successfully from ankle to groin bilaterally (Figure 3). Limb compression therapy was administered subsequently for 1 month using a JOBST^®^ FarrowWrap^®^ 4000 Legpiece and a JOBST FarrowWrap Basic Footpiece (BSN Medical INC., Charlotte, NC, USA).

**Figure 4.**
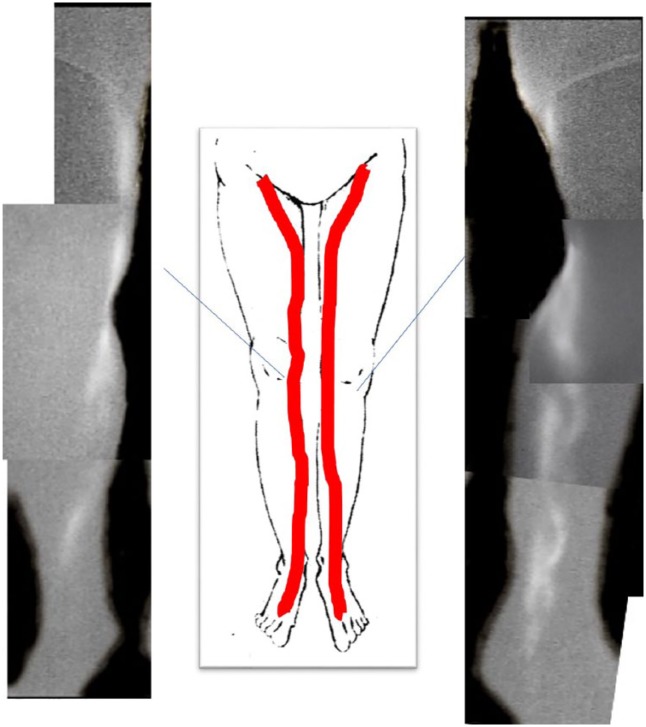
At 3 months after the stripping procedure, lymphatic flow was observed in both legs along the medial side from foot to groin immediately after ICG injection. We judged that lymphatic flow had been restored after the stripping surgery. At 3 h after the injection, the images showed almost the same linear pattern in both lower limbs.

At 3 months after the stripping procedure, lymphatic function was evaluated using ICG imaging to check for deterioration of lymphatic flow.

Lymphatic flow was observed in both legs along the medial side from the foot to the groin immediately after injection of ICG (Supplement Figure 2). At 3 h post-injection, the images showed almost the same linear pattern in both lower limbs. We determined lymphatic flow to have been restored after the stripping surgery. The LEL index was 163 on the right and 162 on the left leg. To date, there has been no deterioration of lymphatic flow.

## Discussion

We have encountered a patient with a combination of venous and lymphatic stasis in whom lymphatic function was improved after surgical treatment for varicose veins.

The pathogenesis of lymphatic dysfunction–related chronic venous insufficiency remains unknown.^14,15^ A deeper understanding of the concept of phlebolymphology is required. Phlebolymphedema is a condition involving a mixture of venous and lymphatic insufficiency.^16^

Venous hypertension caused by venous insufficiency, including varicose veins, triples the flow of lymph and doubles its concentration of fibrinogen, thereby increasing net transport of fibrinogen across the interstitial space by 600%.^17^ However, there is no significant change in the fibrinolytic activity of lymph. The quantity of fibrinogen passing across the interstitial space increases, but fibrinolysis does not increase. Both these changes increase the risk of fibrin being deposited in the tissues, including the veins and lymphatics. When fibrin is deposited around the capillaries, it blocks diffusion of oxygen and leads to tissue fibrosis that is visible as lipodermatosclerosis and necrosis, that is, a venous ulcer.^17^

Lymphatic stasis caused by venous hypertension leads to inflammation of the vein wall by inducing degeneration of adipocytes at that site.^16^ This inflammation causes proliferation of medial smooth muscle cells and excessive production of fibrous matter, leading to hyperplasia, which in turn reduces the elastic compliance of the venous wall.^18,19^ Therefore, structural deterioration of the microlymphatic network might contribute to venous insufficiency.^20^ Furthermore, it has been suggested that sustained inflammation could lead to apoptosis of the lymphatic vasculature. The functions of the venous and lymphatic systems are closely related, so it is thought that many vascular disorders are not simply diseases of the venous system or lymph ducts and that if the function of one declines, then the other will also be affected. Consequently, a vicious cycle develops between lymphatic stasis and venous insufficiency. The dynamics of the venous and lymphatic systems may interact as an inseparable and mutually dependent dual outflow system in the tissues; the mechanism is complex and homeostasis can be maintained between the two systems. Venous treatment modalities including surgery, such as stripping, may be useful for breaking the vicious cycle between lymphatic stasis and venous insufficiency.

The lymphatic vessels may be located too deeply to be observed by ICG lymphography preoperatively because of edema, but could become visible postoperatively following resolution of edema. However, in our case, the LEL index, which indicates severity of edema, was lower in the left leg where lymphatic flow was not observed than in the right lower limb where lymphatic flow was observed. It is plausible that lymphatic flow was improved after stripping of the GSV.

The main limitation of this study is that only superficial lymphatic flow was observed. Further study is needed to observe lymphatic flow in the deep layer using lymphoscintigraphy or magnetic resonance lymphography.

## Conclusion

In this report, we described a patient in whom return of lymphatic function was confirmed by ICG after stripping of the GSV for varicosity. Venous treatment modalities including surgery, such as stripping, may be useful for breaking the vicious cycle between lymphatic stasis and venous insufficiency.
